# Effects of metabolic cancer therapy on tumor microenvironment

**DOI:** 10.3389/fonc.2022.1046630

**Published:** 2022-12-13

**Authors:** Petra Hyroššová, Mirko Milošević, Josef Škoda, Jiří Vachtenheim Jr, Jakub Rohlena, Kateřina Rohlenová

**Affiliations:** ^1^ Institute of Biotechnology of the Czech Academy of Sciences, Prague, Czechia; ^2^ Faculty of Science, Charles University, Prague, Czechia; ^3^ 3rd Department of Surgery, First Faculty of Medicine, Charles University and University Hospital Motol, Prague, Czechia

**Keywords:** cancer, metabolism, tumor micro environment (TME), glycolysis, oxidative phoshorylation, fatty acid metabolism, nucleotide metabolism, endothelial cells

## Abstract

Targeting tumor metabolism for cancer therapy is an old strategy. In fact, historically the first effective cancer therapeutics were directed at nucleotide metabolism. The spectrum of metabolic drugs considered in cancer increases rapidly – clinical trials are in progress for agents directed at glycolysis, oxidative phosphorylation, glutaminolysis and several others. These pathways are essential for cancer cell proliferation and redox homeostasis, but are also required, to various degrees, in other cell types present in the tumor microenvironment, including immune cells, endothelial cells and fibroblasts. How metabolism-targeted treatments impact these tumor-associated cell types is not fully understood, even though their response may co-determine the overall effectivity of therapy. Indeed, the metabolic dependencies of stromal cells have been overlooked for a long time. Therefore, it is important that metabolic therapy is considered in the context of tumor microenvironment, as understanding the metabolic vulnerabilities of both cancer and stromal cells can guide new treatment concepts and help better understand treatment resistance. In this review we discuss recent findings covering the impact of metabolic interventions on cellular components of the tumor microenvironment and their implications for metabolic cancer therapy.

## Introduction

**Figure 1:**
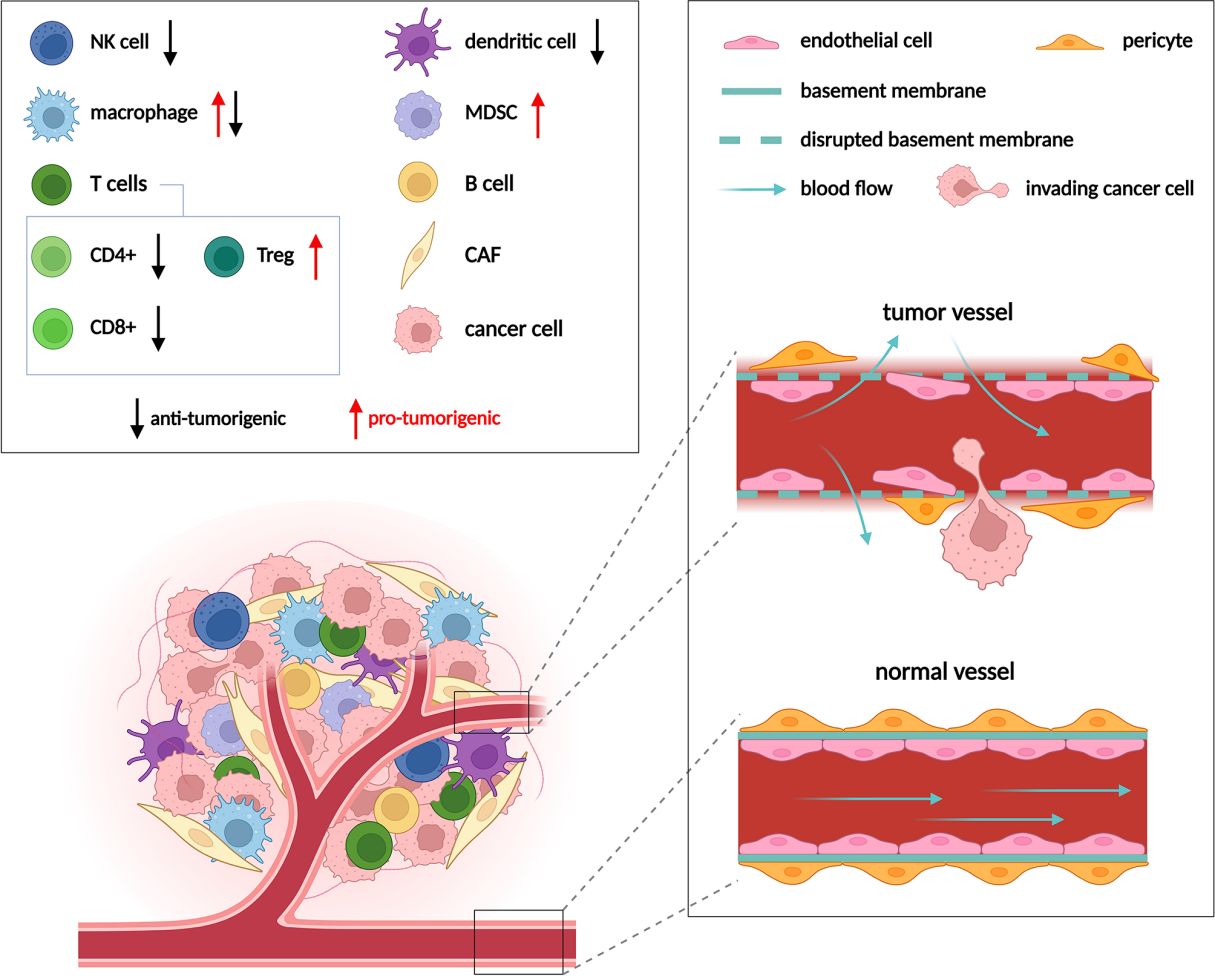
Cell populations in the TME and their role in tumor progression. Composition of the TME varies between different tumor types, but usually includes cancer cells, immune cells, cancer associated fibroblasts (CAFs) and endothelial cells (ECs). Multiple types of immune cells are present in the TME, only those mentioned in following figures are depicted. Immune cells can either promote (red arrows) or suppress (black arrows) tumor growth. ECs form the inner lining of blood vessels. Vessels are normally well ordered, covered by an intact basement membrane and a layer of pericytes. In the TME vessels become disorganized due to excessive pro-angiogenic signaling. Such abnormal vessels are leaky and become a gateway for tumor metastasis. Adapted from “Tumor Microenvironment 2”, by BioRender.com (2022). Retrieved from https://app.biorender.com/biorender-templates.

Metabolic anti-cancer drugs target pathways such as glycolysis, glutaminolysis or oxidative phosphorylation that support proliferation and redox balance in cancer cells. These pathways are fundamental to cellular metabolism in general, and while being crucial for cancer cells, they are not strictly cancer specific. Non-transformed proliferating cells and to some degree also non-proliferating somatic cells engage the very same pathways to perform a number of essential functions (1). Metabolic drugs that target these pathways will thus also impact cell types other than cancer cells when applied *in vivo*. Indeed, besides cancer cells, tumors comprise multiple other non-transformed cell types such as fibroblasts, endothelial cells (ECs) and immune cells that collectively form the tumor microenvironment (TME) (**Figure 1**). Within the TME, cancer cells interact with and alter metabolism of these tumor resident non-transformed stromal cell types on multiple levels. For example, cancer associated fibroblasts (CAFs) upregulate collagen synthesis for extracellular matrix deposition (2, 3), tumor associated ECs stimulate glycolysis to support angiogenesis, and various types of the immune cells such as macrophages or T-cells are modulated by the TME towards immunosuppression. Vice versa, stromal cells may supply cancer cells with metabolites, and cancer cells can obtain nutrients externally from the blood stream (4), which is facilitated by EC-driven angiogenesis.

Strategies to target metabolism of tumor cells do not routinely consider the metabolism of non-cancer cells (e.g., immune cells, ECs and CAFs) within the tumor. However, these additional aspects might be essential when searching for a truly effective and practicable metabolic treatment. Below we summarize how pharmacological modulation of major metabolic pathways in cancer affects individual stromal cell types.

## Glycolysis

**Figure 2:**
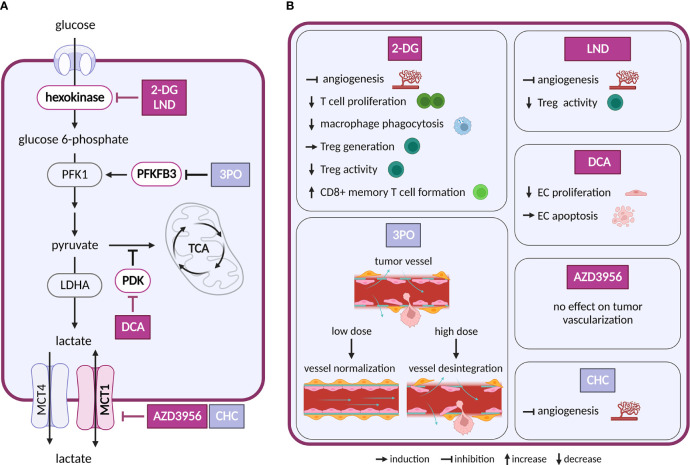
Inhibitors of glycolysis and their effect on TME cell types. **(A)** Schematic representation of glycolysis and lactate exchange. Only inhibitors with a reported effect in the TME are shown. Inhibitors tested in clinical trials for cancer are highlighted in dark violet, inhibitors tested in preclinical studies are shown in light grey-blue **(B)** Effects of the inhibitors shown in **(A)** on cell populations in the TME.

Increased glycolysis is the best known metabolic hallmark of cancer (5, 6). It provides cancer cells with ATP and metabolic intermediates that are channeled into biosynthetic pathways to form nucleotides, amino acids (AAs) and lipids, and to maintain redox homeostasis (7, 8). Pyruvate produced by glycolysis is converted to lactate to sustain high glycolytic flux (9). This conversion is favored because pyruvate import into mitochondria is suppressed by pyruvate dehydrogenase kinase (**Figure 2**). Attenuation of glycolysis in cancer cells will therefore compromise biosynthesis and alter the redox balance. Two main approaches have been pursued to achieve these goals: (i) direct inhibition/modulation of the glycolytic pathway and (ii) interference with the excretion of lactate.

Intervention strategies that inhibit glycolysis are challenging due to its systemic importance. First attempts to target glycolysis by unmetabolizable glucose analog 2-deoxyglucose (2-DG) were unsuccessful, as the treatment caused toxicity associated with hypoglycemia (10). Despite the initial disappointment, other glycolysis inhibitors have been intensively evaluated and several of them were brought into clinical trials (11). The tested drugs target various steps of the glycolytic pathway, such as glucose transporters (GLUTs), hexokinase 2 (HK2), pyruvate dehydrogenase kinase (PDK), lactate dehydrogenase A (LDHA) and monocarboxylate transporters (MCTs).

Besides cancer cells, glycolysis is crucial also in other cell types of the TME. For example, CAFs, an abundant stromal type in solid tumors, display increased expression of enzymes involved in glycolysis and produce lactate (12). Interestingly, redirection of glucose metabolism toward tricarboxylic acid (TCA) cycle in CAFs by silencing PDK4 showed decreased growth of co-injected cancer cells in xenograft models (13).

ECs are glycolytic already at baseline, and their glycolytic flux is further increased in response to tumor-produced vascular endothelial growth factor (VEGF) and fibroblast growth factor (FGF) (14). Both VEGF and FGF upregulate an activator of glycolysis 6-phosphofructo-2-kinase/fructose-2,6-biphosphatase 3 (PFKFB3) in tumor ECs (TECs), while FGF also activates a glycolytic enzyme HK2 (15, 16). PFKFB3 is responsible for the production of fructose-2,6-bisphosphate, an allosteric activator of phosphofructokinase 1 (PFK-1). As heightened glycolysis in ECs promotes angiogenesis, inhibition of PFKFB3 or HK2 genetically or pharmacologically, using 3-(3-pyridinyl)-1-(4-pyridinyl)-2-propen-1-one (3PO) or 2-DG and lonidamine (LND), respectively, impairs vessel formation *in vitro* and *in vivo* (15–19). Interestingly, in the context of tumor angiogenesis inhibition of PFKFB3 by 3PO can have different outcomes, depending on the administered dose. Low dose 3PO, that does not affect cancer cells, leads to normalization of tumor vasculature and consequently to decreased cancer cell invasion and metastasis (20). However, highest tolerable dose of 3PO, used in clinical trials, disrupts TECs metabolism and induces apoptosis, which in turn leads to vessel disintegration and increased metastasis (21). Similar dose-dependence has been observed for dichloroacetate (DCA). This compound, an inhibitor of PDK, promotes shunting of pyruvate into mitochondria, which attenuates aerobic glycolysis. At lower doses DCA decrease proliferation and migration of ECs, but at higher doses it drives ECs to apoptosis (22). It is thus important to routinely evaluate dosing of new glycolytic inhibitors to avoid adverse effects on vessel integrity that might be pro-metastatic.

Glucose is also critical for proliferation and activation of immune cells, such as effector T cells (23), natural killer (NK) cells (24) and M1 macrophages (25). 2-DG decreases phagocytosis of elicited macrophages (26), proliferation of T cells *in vitro* (27) and reduces IFNγ secretion in splenocytes (28). Depletion of glucose in tumor environment by cancer cells limits effector function of metabolically restricted T cells and leads to their functional exhaustion (29, 30). 2-DG treatment promoted generation of regulatory T cells (Treg) cells *in vitro* and *in vivo* in the context of autoimmune disease (31). However, in other study, 2-DG and LND showed opposite results, where it reversed suppressive activity of Treg cells (32). These differences might be caused by different experimental settings or metabolic state of Treg cells and need to be addressed further. Additionally, inhibition of glycolysis with 2-DG promotes formation of long-lived CD8+ T memory cells (33). Overall, considering different metabolic dependencies of immune cell types, it is not surprising that inhibition of glycolysis has different effects on immune response.

The main product of aerobic glycolysis is lactate, an important carbon source not only in the TME, but also during normal physiology (4). Lactate exchange with the extracellular space is maintained by MCT1 and 4 (34). MCT1 allows bi-directional transport that depends on substrate gradient (35). Inhibition of MCT1 is thus expected to interfere with lactate export in highly glycolytic cancer cells, resulting in their elimination. At the same time, this treatment would deprive other cell types of an important carbon source (possibly also inducing cell death). Inhibition or a decrease in expression of MCT1, MCT4 and LDHA in cancer cells slowed down tumor growth (36, 37). Importantly, MCT1 and MCT4 are expressed not only in cancer cells of various origin, but also in stromal cells (35). MCT1 inhibitor AZD3965 underwent Phase I clinical study (NCT01791595).

Several studies suggest a so-called reverse Warburg effect, where CAFs feed cancer cells with lactate or pyruvate that cancer cells oxidize in mitochondria (13, 38, 39). In this setting, silencing of MCT4 in CAFs decreased lactate export from CAFs and decreased growth of tumors *in vivo* (39). Such reduction in CAF-exported lactate would not only deprive cancer cells of an alternative fuel, but also eliminate the immunosuppressive effects of elevated lactate concentrations (see below).

In ECs, lactate uptake through MCT1 activates hypoxia-inducible factor 1 alpha (HIF-1α) and consequently promotes angiogenesis through increased expression of basic fibroblast growth factor and vascular endothelial growth factor receptor 2 (VEGFR2) (40). However, an inhibition of MCT1 showed different outcomes with two different compounds: a-cyano-4-hydroxycinnamate (CHC) decreased angiogenesis in tumors (40), whereas AZD3965 showed no effect on tumor vascularization in a murine model. These differences might be caused by unspecific effects of CHC (41).

Lactate negatively affects function and survival of T cells and NK cells in the TME, which leads to immune escape. In line with this, lower lactate production in melanoma tumors with downregulated LDHA expression increased infiltration of T and NK cells, which slowed down tumor growth (42). Lactate is also known to promote M2-like polarization of tumor-associated macrophages (TAMs) that promote tumor progression by production of immunosuppressive cytokines (43, 44). Suppression of lactate production in cancer cells lowered the lactate effect on M2 macrophage polarization (45, 46). Of note, in T cells MCT1 inhibition was considered as an immunosuppressive therapy. While MCT1 inhibitors can block T cell proliferation, this does not interfere with their effector function and viability (47).

In summary, while complete inhibition of glycolysis will reduce viability/proliferative potential of tumor cells, it will also suppress anti-tumor immune responses and lead to tumor vessel disintegration that promotes metastasis, rendering treatment ineffective. Partial inhibition of glycolysis might thus result in a more favorable outcome *in vivo*. On the other hand, inhibition of lactate transporters seems to have less negative effect on the vasculature and immune responses within the TME.

## Oxidative phosphorylation

**Figure 3:**
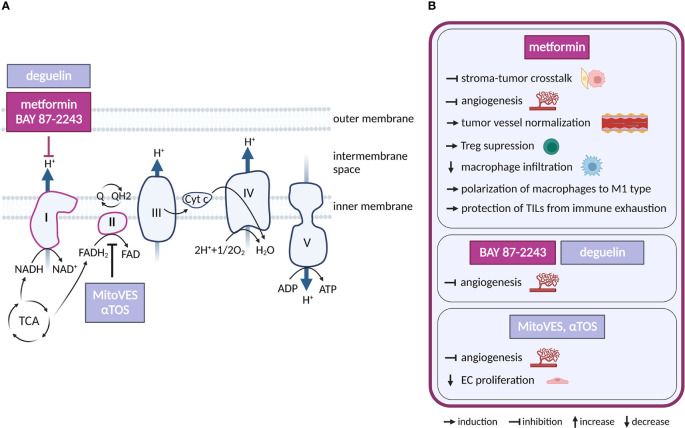
Inhibitors of oxidative phosphorylation and their effects on TME cell types. **(A)** Schematic representation of the OXPHOS system. Only inhibitors with a reported effect in the TME are shown. Inhibitors tested in clinical trials for cancer and in clinical practice are highlighted in dark violet, those in preclinical studies are in light grey-blue. **(B)** Effects of the OXPHOS inhibitors shown in **(A)** on cell populations present in the TME.

Oxidative phosphorylation (OXPHOS) is a system of five respiratory complexes at the inner membrane of mitochondria that is best known for ATP production (**Figure 3**). However, in proliferating cancer cells ATP generation by OXPHOS is dispensable, and OXPHOS maintains intracellular redox balance to enable synthesis of AAs aspartate and asparagine as well as *de novo* synthesis of nucleotides (48–51). OXPHOS activity is often but not always reduced in cancer cell lines and tumors, as an outcome of lower mitochondrial DNA (mtDNA) content or presence of mtDNA mutations (52). Still, even low levels of OXPHOS activity can support biosynthesis and promote primary tumors (53). Interestingly, elevated OXPHOS activity marks drug-resistant and persistent populations of cancer cells (54), and some cancer types upregulate OXPHOS (52, 55), such as those localized in well perfused and oxygenated areas (56, 57). Thus, OXPHOS inhibitors are considered for cancer therapy as they interfere with biosynthesis and upregulate reactive oxygen species (ROS) production from the respiratory chain (58, 59).

Regarding potential cancer therapeutics, respiratory complex I (CI) is the most frequent target. Multiple CI inhibitors with anti-cancer properties have been developed and/or tested, including IACS-010759, BAY 87-2243, tamoxifen, deguelin and the anti-diabetics metformin and phenformin (60–65). Complex II (LND, αTOS) (66, 67), Complex III (atovaquone, adaphostin) (68, 69), complex IV (arsenic trioxide) (70) or combined CI/CII inhibitor Mitotane (71) have also been considered for therapy. OXPHOS-directed compounds have been chemically modified for specific delivery into mitochondria and increased efficacy, as demonstrated by mitochondrial-targeted tamoxifen (MitoTam, CI inhibitor) (72), mitochondria-targeted metformin (MitoMet) (73, 74) and mitochondria-targeted αTOS (MitoVES) (75). Several OXPHOS-directed compounds entered clinical trials in cancer. The best developed of these is metformin, which is routinely used for the treatment of diabetes. In addition, IACS-010759 (NCT02882321, NCT03291938) and MitoTam (MitoTam-01 trial; EudraCT 2017-004441-25) are in clinical trials, while a trial with BAY 87-2243 (NCT01297530) has been terminated.

Metformin is the most studied OXPHOS inhibitor in the clinical settings and its effects on the TME are also the best characterized. Metformin was associated with a reduced risk of cancer (60), and a later prospective study (10 years, 1353 patients) showed that metformin in diabetic patients reduces cancer mortality by 43% (76). The related but more potent CI inhibitor phenformin has been withdrawn due to lactic acidosis complications (77, 78). Metformin has pleiotropic effects (CI, adenosine monophosphate-activated protein kinase (AMPK), etc.), but its anti-tumor efficacy has been linked to CI inhibition in cancer cells (79). Interestingly, by inhibiting CI, metformin synergizes with MCT inhibitors to suppress utilization of external lactate (80), which can be produced by CAFs (44, 81).

CAFs promote migration and invasiveness of cancer cells, including those of pheochromocytoma origin (82, 83). Interestingly, metformin treatment of pheochromocytoma/CAFs co-cultures reduced CAF-induced-migration and invasiveness of succinate dehydrogenase subunit B (SDHB)-deficient pheochromocytoma cells grown as spheroids. The underlying mechanism is unclear at present, but likely involves a direct action of metformin on CAFs, as CAFs with diminished mitochondrial oxidative metabolism showed reduced ability to stimulate migration of pheochromocytoma cells (84, 85). Metformin was shown to upregulate AMPK signaling and thus downregulate HIF-1α, transforming growth factor-β (TGF-β) and interleukin 8 (IL-8) in co-culture of CAFs with human breast cancer lines and thus preventing tumor-stroma crosstalk (86). Metformin also decreased production of pro-tumorigenic cytokine IL-6 *via* NFκB signaling in CAFs cultured with ovarian cancer cell line and upregulated calmodulin−like protein 3 (Calml3) in cultured CAFs that inhibits gastric cancer cell growth (87, 88). Accordingly, metformin may disrupt pro-tumorigenic communication in the TME by hitting both cancer cells and CAFs.

ECs require OXPHOS for angiogenesis and to provide stress resistance in mature vessels (89–91). Multiple OXPHOS-targeting compounds effective against cancer have an anti-angiogenic activity. Complex II inhibitors αTOS and MitoVES (92, 93), as well as arsenic trioxide derivative GSAO (94) inhibit tumor angiogenesis by elevating intracellular ROS which eliminates proliferating ECs. Inhibition of angiogenesis by metformin has been linked to suppression of HIF-1α *via* AMPK signaling (95, 96), or to inhibition of platelet-derived growth factor β (PDGF-β) that lead to normalization of tumor vasculature (97). Given the pleiotropy of metformin action, it has not been conclusively demonstrated that its anti-angiogenic effect is directly linked to CI inhibition. Still, unrelated, and presumably more specific CI inhibitors BAY 87-2243 (63) and deguelin (98) also inhibit angiogenesis, suggesting that CI inhibition plays a causal role in this effect.

In the immune compartment, OXPHOS supports function of pro-tumorigenic Tregs (99), suggesting that OXPHOS inhibition could improve anti-tumor immune responses. In line, metformin treatment suppresses Tregs in tumors (100) and CI inhibitor MitoTam improves efficacy of immune checkpoint therapy in a pre-clinical renal cancer model (101). Metformin lowers macrophage infiltration in tumors and skews TAM polarization from M2 to anti-tumor M1 phenotype (102, 103). Metformin also protects tumor infiltrating lymphocytes (TILs) from immune exhaustion and apoptosis, supporting tumor rejection in immunocompetent mice, but not in immunodeficient SCID mice (104). Mechanistically, metformin treatment has been shown to promote aberrant glycosylation of programmed death ligand-1 (PD-L1) thus reducing its levels on cancer cells and blocking the PD-L1/PD-1 signaling (105).

Taken together, OXPHOS inhibition may be effective against tumors *via* direct targeting of cancer cells, inhibition of CAFs, suppression of angiogenesis and by boosting the immune response. However, not all these aspects have been conclusively demonstrated in *in vivo* tumor models.

## Tricarboxylic acid cycle

**Figure 4:**
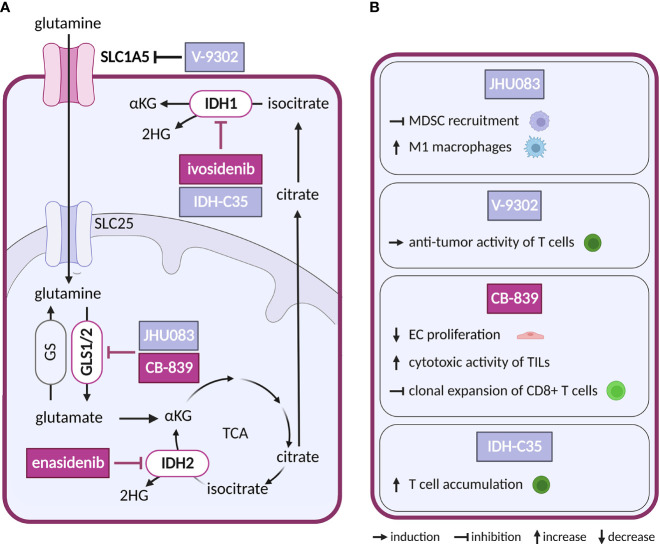
Inhibitors of glutaminolysis or IDH1/2, and their effects on TME cell types. **(A)** Schematic representation of glutaminolysis and IDH1/2 reactions. Inhibitors of glutamine metabolism with a reported effect in the TME and all inhibitors of IDH1 and IDH2 mentioned in the text are shown. Inhibitors tested in clinical trials for cancer are highlighted in dark violet, while those tested in preclinical studies are shown in light grey-blue. **(B)** Effects of the glutamine metabolism inhibitors and IDH-C35 shown in **(A)** on cell populations present in the TME.

The TCA cycle (**Figure 4**) connects cellular metabolism to oxidative phosphorylation and contributes to both catabolism and anabolism. Multiple cancers harbor mutations or deletions in TCA cycle enzymes isocitrate dehydrogenase (IDH), fumarate hydratase (FH), succinate dehydrogenase (SDH) and succinyl-CoA ligase (SUCL) (106–110). As the TCA produces metabolic intermediates with inhibitory effects on enzymes that remove epigenetic marks from the chromatin (6), these alterations affect epigenetics and promote transformation and progression of tumorigenesis.

So far only IDH1/2-mutated enzymes have been targeted pharmacologically in cancer, while no direct inhibitors are available for FH, SDH or SUCL loss-of-function malignancies. Gain-of-function mutations in isoforms IDH1 and IDH2 (but not in the main TCA isoform IDH3) leading to production of oncometabolite (R)-2-hydroxygultarate (2HG) instead of physiologically produced α-ketoglutarate (αKG) have been found in hematological and solid tumors (111). Increased levels of 2HG inhibit αKG dependent oxygenases which through epigenetic dysregulation promote oncogenesis (112). Small molecule inhibitors of mutant IDH2 and IDH1 are in clinical use in acute myeloid leukemia, including enasidenib (AG-221) and ivosidenib (AG-120). Showing potency both *in vitro* and *in vivo*, enasidenib has been in clinical trials in several other types of cancer with mutated IDH2 (NCT02273739, NCT02577406, NCT01915498) (113, 114). With respect to the TME, there are conflicting reports as to the effect of IDH1/2 mutation in cancer cells on the ECs, showing both increased (115, 116) and reduced pro-angiogenic signaling (117). In addition, in intrahepatic cholangiocarcinoma IDH mutations were linked to reduced immune response (118, 119). Reductions in chemokines and suppression of T cell accumulation were reversed by IDH-C35, a specific inhibitor of mutant IDH1 (120). As mutant IDH1/2 are restricted to cancer cells, this effect is very likely secondary to reduction of the oncometabolite. Notably, scRNA-seq analysis of two types of glioblastoma harboring the same IDH1 mutation, astrocytoma and oligodendroglioma, revealed shared developmental hierarchy and common progenitors for all analyzed gliomas, while the TME composition differed between the two tumor types, which is most likely driven by the distinct genetic background (121).

## Glutaminolysis

Glutamine, a non-essential AA, is utilized by cancer cells for biomass synthesis and as a source of energy. To meet the increased metabolic needs of rapid proliferation, glutamine is supplied through blood at a concentration as high as 0.5 mM (122). Exogenous glutamine is transported into the cell through solute carrier family 1 member 5 (SLC1A5) but can also be synthesized endogenously by glutamine synthase (GS) in some cell types (**Figure 4**). Glutamine is further metabolized into glutamate by glutaminases (GLS1/2). Glutamate can be converted to αKG by glutamate dehydrogenase (GLUD) or serve as a nitrogen donor to produce other AAs as well as glutathione for antioxidant defense (123, 124). *Via* αKG glutamine provides TCA cycle intermediates in a process also known as anaplerosis, which can support proliferation in nutrient-restricted environments (123, 124).

Given the ubiquitous role of glutamine as a substrate, it is not surprising that glutamine utilizing reactions are targeted for anticancer treatment. Mimetic compounds, such as 6-diazo-5-oxo-L-norleucine (DON), bind covalently to the active sites of multiple glutamine-dependent enzymes. DON and other glutamine mimetics, acivicin and azaserine, were explored as potential anticancer treatment (125), but their success is limited by high toxicity. To avoid this toxicity, prodrugs of DON were synthetized to release the active payload at specific sites (126). The prodrug DRP-104 (sirpiglenastat) is being tested in an ongoing clinical trial in advanced stage solid tumors as a single treatment and in combination with anti-PD-L1 antibody (atezolizumab) (NCT04471415). Blocking of glutamine metabolism using DON derived prodrug, JHU083, in the TME has not only directly impacted cancer cells, but also acted on the immune compartment. It inhibited recruitment of myeloid derived suppressor cells (MDSCs) and increased generation of antitumor inflammatory TAMs, leading to decreased metastatic potential and improved anti-tumor immunity (127, 128).

In glutamine deprived conditions within the TME, CAFs can increase glutamine synthesis to provide glutamine to cancer cells (129). Thus, targeting glutamine uptake into cancer cells by inhibitors of SLC1A5, such as V-9302, would interrupt such communication. This approach has been explored experimentally in preclinical studies with triple negative breast cancer. Intriguingly, V-9302 suppressed glutamine uptake in cancer cells but not in CD8+ T cells, as T cells were able to upregulate an alternative glutamine transporter. Hence, glutamine availability for T cells increased, stimulating glutathione synthesis in T cells and improving their antioxidant status and anti-tumor activity (130, 131).

As an alternative to glutamine mimetic compounds, researchers have targeted the first enzyme of the glutaminolysis pathway, GLS, which is frequently upregulated in cancer (132). Several allosteric inhibitors have been designed to target isoforms of GLS, such as CB-839 (telaglenastat) and bis-2-(5-phenylacetamido-1,2,4-thiadiazol-2-yl)-ethyl sulfide (BPTES) (133, 134). CB-839 has been tested both as a single treatment and in combination with antimetabolites and immunotherapy (azacytidine, nivolumab), in several types of cancer (NCT02071927, NCT02771626). For example, a combination with glutamine uptake inhibitor V-9302 overcame resistance of hepatocellular carcinoma, a glutamine dependent cancer type otherwise resistant to CB-839 alone (135). Besides the direct effect on cancer cells, CB-839 improved the cytotoxic activity of TILs. By altering glutamine metabolism in cancer cells, CB-839 increased extracellular glutamine levels available to T cells for GLS independent activities such as antigen activation (136). On the other hand, in lung adenocarcinoma CB-839 had an opposite effect on CD8+ T cells, inhibiting their clonal expansion and activation induced by immunotherapy (137). GLS may also be an interesting target in ECs, where it supports proliferation that is reduced by CB-839 treatment (138).

BPTES has been used for targeting KRAS-mutated cancer cells that are addicted to glutamine. Administration of BPTES inhibited tumor growth in Burkitt lymphoma model, through metabolic alteration of glutamate, αKG, succinate and fumarate while additionally increasing ROS levels (139). Consistent with GLS1 supporting pathological angiogenesis, impairment of angiogenesis was observed in ECs treated with a combination of BPTES and a PDK inhibitor DCA (22, 138). BPTES also upregulated PD-L1 on cancer cells, which interfered with T cell activity. This was mitigated by co-treatment with anti-PD-L1 antibody, which rescued T cell function (140). In recent years there has been effort to utilize more specific approaches to BPTES delivery using nanoparticles to avoid systemic and off target effects (141).

GS has been shown as critical for EC motility and migration, processes that underlie angiogenesis during development and in pathological states, making GS an attractive target for inhibition of pathological angiogenesis (142). Furthermore, genetic deletion of GS in TAMs decreases glutamine in TME, which results in tumor vessel normalization, reactivated T cells and decreased cancer cell motility (143).

Overall, these results suggest that more specific targeting and combination therapy might be necessary to overcome systemic toxic effect of glutamine deprivation therapy as well as complex metabolic crosstalk present in TME.

## Amino acids

Metabolism of proliferating cancer cells may become dependent also on other AAs that are normally non-essential, providing therapeutic opportunities in reducing AA serum levels (144). The first AA targeted in this manner was asparagine. An enzyme called L-asparaginase (Ciderolase, Erwinase), originally identified in guinea pig serum (145, 146), can degrade blood asparagine and hereby limit its availability. L-asparaginase is effective because the enzyme asparagine synthase (ASNS, responsible for endogenous asparagine synthesis) is decreased in some cancer cells, making them dependent on exogenous asparagine for proliferation (147, 148). At present, L-asparaginase is an approved therapy in ALL and non-Hodgkin lymphoma, while in other leukemias and solid cancers it is in clinical trials (NCT03665441, NCT01574274). Beside cancer cells, L-asparaginase treatment may also affect other cell types in the TME. ECs utilize asparagine for sprouting and vessel growth, particularly under glutamine deprivation (138), while CD8+ T cells need extracellular asparagine for optimal immune response (149), hinting at a possible complex response to asparagine depletion in the TME.

Arginine is synthetized through subsequent reaction of argininosuccinate synthase (ASS) and argininosuccinate lyase (ASL). Interestingly, a subset of malignancies do not express ASS, opening a potential therapeutic window for arginine deprivation therapy using arginine-degrading enzymes arginine deiminase (ADI) (150, 151) or arginase (152). ADI has progressed into phase 3 trials in cancer, both as a single agent and a combinatorial therapy (NCT02709512, NCT05317819). In the TME arginine depletion impaired T cell responses. Chimeric antigen T cells (CAR-T) proliferation was rescued by expression of the ASS and ornithine transcarbamylase (OTC) without loss of cytotoxicity of CAR-Ts, suggesting that reengineered cells prevail in arginine depleted environment (153). In addition, extracellular arginine shifts T cell metabolism from glycolysis to oxidative phosphorylation, priming them for activation and generation of memory cells (154). To locally increase arginine availability to T cells, an E. Coli strain was engineered that converts ammonia to arginine for enhanced efficacy of immunotherapy. Arginine level in tumor homogenates was increased following intratumoral injection of engineered E. Coli, the number of TILs was elevated and there was a synergistic effect with PD-L1 blocking therapy (155).

Methionine is an essential AA, still the dependence of cancer cell on methionine is particularly pronounced (156). Unlike normal cells, proliferating cancer cell are unable to utilize/recycle methionine precursor homocysteine (157). Several approaches were developed to decrease serum levels of methionine, including the enzyme methionase and dietary restrictions (158). While depleting methionine from serum proved challenging to strike the balance between toxicity and benefits, recently there was a reemergence of this approach (159, 160). With respect to TME, in hepatocellular carcinoma patients increased excretion of methionine metabolism intermediates from cancer cells induced T cell exhaustion due to epigenetic changes, suggesting that reducing methionine availability to cancer cells could stimulate immune response (159). On the other hand, low levels of methionine in the TME, due to excessive consumption by cancer cells, also reduced anti-tumor immunity *via* epigenetic changes in T cells (160). Thus, selective inhibition of methionine uptake by cancer cells but not immune cells, similarly as previously shown for glutamine (130), might be the most effective way to preserve and boost anti-tumor immunity.

Proline is a non-essential amino acid. Nevertheless, proline synthesis enzymes are frequently upregulated in cancer (161, 162) and endogenous proline synthesis supports cancer cell proliferation. While exogenous proline can be obtained from the TME, *de novo* proline synthesis in cancer cells is required to maintain cellular redox state *via* NADH recycling by 5-carboxylate reductase 1 (PYCR1) (163). On the other hand, proline degradation by proline dehydrogenase (PRODH) sustains breast cancer metastases and promotes epithelial to mesenchymal transition in lung cancer, effects sensitive to PRODH inhibitor L-tetrahydro-2-furoic acid (L-THFA) (164, 165). Proline synthesis, both in cancer cells and in CAFs, modulates the TME. PYCR1 in cancer cells in conjunction with the mitochondria protease Lon elicits ROS-dependent production of pro-inflammatory cytokines that promote M2 macrophage polarization and angiogenesis (165). Proline synthesis in CAFs then enables production and deposition of collagen, a major component of the extracellular matrix. Suppression of PYCR1 in CAFs inhibits collagen deposition, tumor growth and metastatic potential in breast cancer (3). Consistently, autophagy deficiency in CAFs impedes proline biosynthesis and collagen deposition (166). Inhibitors of proline metabolism have not yet been evaluated in clinical trials. However, PYCR1 expression in CAFs is epigenetically regulated and depends on cytosolic acetyl-CoA (3). Besides PYCR1 inhibitors that are under development (167), more established agents that interfere with acetyl-CoA management (inhibitors of histone acetyl transferase EP300 or ATP citrate lyase) could be considered for therapy.

## Fatty acid metabolism

**Figure 5:**
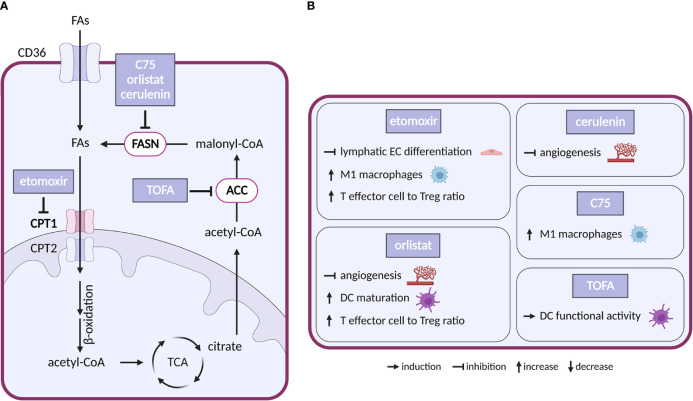
Inhibitors of fatty acid metabolism and their effects on TME cell types. **(A)** Schematic representation of FA metabolism. Only inhibitors with a reported effect in the TME are shown. Note that none of these inhibitors entered clinical trial in cancer. **(B)** Effect of the inhibitors shown in **(A)** on cell populations present in the TME.

Fatty acid (FA) metabolism (**Figure 5**) is important for proliferation and survival of cancer cells under conditions of glucose limitation, where FA β-oxidation (FAO) serves as an alternative energy source (168). Indeed, upregulation of a key enzyme in FAO, carnitine O-palmitoyltransferase 1 (CPT1) protects cancer cells in glucose-deprived conditions and CPT1 knockdown sensitizes cells to therapy (169). Conversely, overexpression of lipogenic enzymes such as acetyl-CoA carboxylase (ACC) and fatty acid synthase (FASN) is commonly seen in tumors and linked to poor prognosis (168).

Inhibitors of enzymes of lipid metabolism (FASN, ACC, CPT1) have been intensively studied and several compounds have shown efficacy in experimental settings: (i) With respect to FASN inhibitors, orlistat, a clinically used anti-obesity drug (a pancreatic lipase blocker), shows anticancer effectivity in multiple cancer models (170). Cerulenin and its synthetic analogue C75 slowed down the growth of breast and ovarian cancer xenografts (171, 172). Besides that, number of new-generation FASN inhibitors have been developed recently including TVB-2640, which is now in clinical trials in cancer (NCT02223247). (ii) Regarding ACC inhibitors, 5−tetradecyloxy−2−furoic acid (TOFA) inhibited the growth of ovarian tumor xenograft *via* downregulation of cell-cycle regulating proteins (173). (iii) For CPT1 inhibitors, etomoxir decreased growth of prostate cancer and leukemia xenografts in mice (174, 175). However, the drug has been discontinued in phase II clinical trial for treatment of heart failure due to hepatotoxicity (176). Another CPT1 inhibitor, ST1326, prevented formation of B-cell lymphoma in mice (177) and CPT1 inhibitor ranolazine administered together with pyruvate dehydrogenase kinase inhibitor DCA inhibited growth of glioblastoma in mice (178).

In the TME, CAFs often feature elevated lipid biosynthesis and upregulation of FASN expression, which promotes lipid transfer from CAFs to cancer cells and stimulates tumor growth (179, 180). Silencing of FASN in CAFs reduced migration of colorectal cancer cells grown in CAF-conditioned media (181). Interestingly, CPT1 was downregulated in metastatic colorectal cancer, but upregulated in the corresponding CAFs. Consistently, in mice DLD1 colorectal cancer cells formed bigger tumors when injected with CAFs overexpressing CPT1A compared to normal CAFs. It appears that in this arrangement the preferential use of FAs by CAFs saves glucose for cancer cells. Pharmacological inhibition of overexpressed CPT1A with etomoxir resulted in reduced tumor growth (182).

Vascular ECs consume FAs as a carbon source for DNA (but not RNA) synthesis. Loss of CPT1 in vascular ECs impairs angiogenesis (183) because of a proliferation (but not migration) defect. CPT1 loss in lymphatic ECs alters epigenetics due to reduced coenzyme A levels and impairs lymphangiogenesis (184). In the adult vasculature, FAO supports vascular integrity (185, 186). Similarly, disruption of FA synthesis (by FASN deficiency) results in defects in angiogenesis and increased vessel permeability linked to decreased palmitoylation, reduced activity of endothelial nitric-oxide synthase, increased malonylation and reduced activity of the mTORC complex 1 (mTORC1) (187). Pharmacological modulation of FA metabolism in ECs recapitulated these effects. CPT1 blockade with etomoxir inhibited lymphatic EC development and differentiation (184). FASN inhibitors orlistat and cerulenin inhibited angiogenesis and slowed down the growth of B16-F10 melanoma tumors in mice (188, 189).

In the immune compartment, FA metabolism regulates differentiation of TAMs. FAO is upregulated in alternatively-activated macrophages that tend to be immunosuppressive (190). FAs, and in particular polysaturated FAs, are abundant in TME, and palmitate or oleate treatment stimulates transition of macrophages towards the immunosuppressive M2 phenotype (191–193). This effect is abolished by the CPT1 inhibitor etomoxir (193), which also promotes the pro-inflammatory M1 phenotype (194). Similarly, linoleic acid, *via* lipid-sensing peroxisome proliferator–activated receptor (PPAR) β/δ signaling, imparts TAMs with pro-tumorigenic properties (195). Interestingly, the FASN inhibitor C75 has inhibited lipid droplet formation and stimulated M1 phenotype *via* MEK1/2 axis in Raw264.7 macrophage cell line (194). These results indicate that the FAs of the TME shape TAM’s phenotype and suggest that pharmacological targeting of FAs could be used to promote anti-tumor immunity.

FA metabolism can also determine the fate of T cells and change their repertoire. While CD8+ effector T cells restrict tumorigenesis by attacking cancer cells, Tregs promote tumorigenesis by suppressing the effector T cells (196). Free FAs are toxic for effector T cells in high concentration, but they promote Tregs whose metabolism favors FA oxidation (197). Thus, while generally TME might accentuate Tregs over T effectors, the inhibition of FA biosynthesis might have opposing effects. Indeed, breast cancer cells release oleate which compromises T effectors (198), and etomoxir treatment resulted in slower growth of glioblastoma in mice and increasing T effector to Treg ratio (199). Interestingly, inhibition of FAO with etomoxir or perhexiline enhanced glycolysis in T effectors in obesity-linked breast cancer, which resulted in reduced breast mammary tumor growth in obese mice (200). These results are further confirmed by a recent study of PPARγ signaling in T effectors, where PPARγ agonist bezafibrate induced mitochondrial respiration and FAO, which improved survival of effector T cells in FA rich TME (201).

Unlike for most other metabolic treatments, the effects of inhibiting FA metabolism were described in dendritic cells (DCs). DCs are important for establishing the immune response as they present antigens to T cells. Lipid accumulation resulted in dysfunction of DCs in antigen presentation (196). Inhibiting FA biosynthesis with ACC inhibitor TOFA suppressed melanoma tumor growth in mice and improved ability of DCs to stimulate T cells (202). Complex recent study showed that inhibition of FA biosynthesis with FASN inhibitor orlistat also resulted in higher T effector and reduced Treg tumor infiltration, increased DC maturation and slowed down tumor growth (203). FAs could potentially also dampen the innate anti-tumor immune response, namely its most important effectors, the NK cells. In lipid rich TME, NK cells tend to accumulate and store lipids to prevent lipid toxicity, but when this intracellular lipid accumulation becomes excessive, it can disrupt proper NK cell function (196).

## Nucleotides

**Figure 6:**
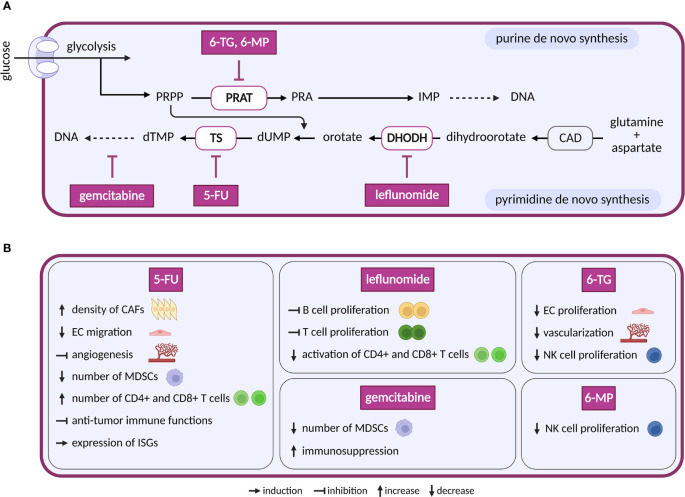
Inhibitors of nucleotide metabolism and their effects on TME cell types. **(A)** Schematic representation of nucleotide *de novo* synthesis. Anti-nucleotide therapy compounds with a reported effect in the TME are shown. These compounds are in clinical trials for cancer or in clinical use. **(B)** Diverse effects of the nucleotide inhibitors shown in **(A)** on cell populations present in the TME.

In highly proliferating cells, purine and pyrimidine nucleotides are indispensable for nucleic acid synthesis. *De novo* synthesis of nucleotides (**Figure 6**) requires input from pentose phosphate pathway, TCA cycle, OXPHOS and one-carbon pool (204). Energetically less demanding salvage pathways, that recycle intracellular or extracellular nucleoside pools, are generally preferred in differentiated cells (205), although preferential use of salvage pathways has also been observed in breast cancer during epithelial–mesenchymal transition (206).

The first drug to target *de novo* nucleotide synthesis, aminopterin, was successful in patients with acute lymphoblastic leukemia. Aminopterin is a folate analog that inhibits dihydrofolate reductase-dependent synthesis of tetrahydrofolate (207). Tetrahydrofolate is essential in the production of purines and pyrimidines, and its deficiency results in lower DNA, RNA and protein synthesis. Newer derivates of aminopterin, such as methotrexate, trimetrexate and pemetrexed, are called by a common name antifolates (208). The initial clinical success of antifolates led to the development of a new class of drugs called antimetabolites. Antimetabolites are analogues of purines and pyrimidines that interfere with proliferation in two different ways: by inhibition of enzymes involved in nucleotide base synthesis and by incorporation into DNA or RNA (209, 210). Several antimetabolites are currently approved for cancer treatment. The purine analogs 6-mercaptopurine (6-MP) and 6-thioguanine (6-TG) inhibit phosphoribosyl pyrophosphate amidotransferase, the first enzyme in *de novo* purine biosynthesis (209). The pyrimidine analog 5-fluorouracil (5-FU) and its prodrug capecitabine inhibit thymidylate synthase and therefore block synthesis of dTTP needed for DNA replication. Cytarabine and gemcitabine are analogues of pyrimidine deoxycytidine. Both incorporate into the DNA and stop DNA replication (211).

Compounds that directly target *de novo* synthesis of pyrimidine are also available. The most druggable enzyme of pyrimidine synthesis is dihydroorotate dehydrogenase (DHODH) (212), and multiple DHODH inhibitors have been designed, including brequinar, leflunomide, teriflunomide, ASLAN003, BAY2402234 and several others (213). While effective in preclinical mouse models of small cell lung cancer (214), breast cancer, pancreatic cancer and glioblastoma (215, 216), clinical trials in human cancer were less successful. Possibly, cancer cells may overcome nucleotide shortage *via* salvage pathway (217). However, dipyridamole, an inhibitor of nucleoside transport *via* solute carrier family 29 member 1 (SLC29A1/ENT1), showed synergistic effect with brequinar *in vitro*, but not *in vivo* (218). This might be due to rescue by uridine in plasma that can rebound after the use of brequinar (219).

Nucleotide-based therapy has satisfactory outcomes, but its general anti-proliferative effects lack selectivity. Consequently, it also affects cells of bone marrow, intestine and hair follicles, which leads to toxicity (220). Many cells of the TME also have a proliferative character and depend on nucleotides for DNA/RNA synthesis. TECs, for instance, enhance nucleotide synthesis and upregulate several enzymes required for purine and pyrimidine synthesis (20, 221). 5-FU and 6-TG decrease migration and/or proliferation of primary ECs and *in vivo* angiogenesis. Inhibition of pyrimidine synthesis by 5-FU affects migration capacity of ECs stimulated by VEGF and FGF-2, tube formation stimulated by VEGF (222) and angiogenesis induced by murine renal cell carcinoma in dorsal air sac assay (223). Similarly, purine analogue 6-TG inhibited fetal bovine aortic EC proliferation upon VEGF and FGF-2 stimulation. Consistently, increased vascularization of bone marrow in patients with AML was reverted under 6-TG treatment (224) and inhibition of pathways feeding into nucleotide synthesis in ECs such as FAO (183), glutaminolysis (138) and glycolysis (20) diminished proliferation. These effects on ECs might lead to decreased vascularization and subsequently to slower tumor growth.

Compared to ECs, CAFs are less well studied regarding consequences of nucleotide synthesis inhibition. Interestingly, increased density of CAFs was observed following treatment with maximum tolerated dose of 5-FU *in vivo* (225) and in patients with combined treatment of 5-FU and radiotherapy (226).

In the immune compartment, a negative effect on patient immune response is expected, as activated T and B cells metabolically depend on nucleotide synthesis for their proliferation (227–229). Leflunomide, the most clinically developed inhibitor of pyrimidine *de novo* synthesis, blocks proliferation of antibody producing B cells (229), malignant B cells (230), T cells (231) and interferes with activation of CD4+ T and CD8+ T cells in HIV-1 infected patients (232). In a murine autoimmune disease model, leflunomide suppressed B and T cells. Uridine supplementation was able to rescue activity of suppressed cells with the exception of CD8+ T cells (233). As supplementation with exogenous uridine normally compensates for defects/inactivity of *de novo* pyrimidine synthesis, this points to possible off target effects of leflunomide, such as inhibition of MAPK or tyrosine kinases (234). Hence, newer, and more specific inhibitors of DHODH might provide better outcomes. Interestingly, one dose treatment 5-FU reduced the number of MDSCs and therefore activated immune response by stimulating CD8+ T cells (235). Additionally, 5-FU treatment elevated the number of tumor-associated CD4+ and CD8+ T cells (236). However, another study documented impaired anti-tumor immune functions with more 5-FU treatment cycles (237). Similar to pyrimidines, inhibitors of purine metabolism also negatively impact immune cells. 6-TG and 6-MP decreased proliferation of NK cells from peripheral blood (238). Elevated purine levels in proliferating cells increase expression of MHC class I polypeptide-related sequence and in this way stimulate NK group 2D effectors to recognize abnormal cells (239). Notably, antimetabolites are also used as immunosuppressants (240), underscoring their potential to dampen immune response. Surprisingly therefore, they can also support protective immunity. Similar to 5-FU, one-time treatment of mice bearing large tumors with gemcitabine, an analog of pyrimidine deoxycytidine, depleted MDSCs in the spleen (241, 242). However, repeated dose of gemcitabine promoted immunosuppression in the TME (243). Additionally, in an *in vitro* model of viral infection (243) and mouse embryonic fibroblasts (244), inhibition of *de novo* pyrimidine synthesis induced expression of interferon-stimulated genes (ISGs) that are known to promote innate immune response. Current evidence points to predominantly suppressed immune response upon inhibition of nucleotide synthesis. However, these negative effects on immune response can be exploited for therapy. For example, pemetrexed promotes immune checkpoint blockade through *per se* undesired transcriptional activation of PD-L1 in cancer cells, and activation of T-lymphocytes was observed *in vitro* when pemetrexed was combined with the anti-PD-1/PD-L1 therapy (245).

## Conclusion

Metabolic adaptations that promote proliferation and survival of tumor cells represent an attractive target for cancer therapy. However, such metabolic therapy is challenged by the intrinsic flexibility and heterogeneity of cancer metabolism, the narrow therapeutic window due to an overlap with the metabolism of healthy cells (246), and by the complex interactions of metabolic agents in the TME. The available information about how TME responds to metabolic treatments is often limited, indirect and derived from sub-optimal experimental models. The emerging patterns nevertheless suggest that metabolic treatments could substantially impact the TME both to enhance or reduce the efficacy of the intended therapeutic interventions. Although we consider it unlikely that unfavorable interactions in the TME would lead to an outright acceleration of primary tumor growth, they might reduce effectiveness, prevent a lasting remission, and perhaps increase the risk of metastasis.

With respect to the major TME cell types, the immune compartment seems to be the most affected, and is often impacted in a negative manner. Immune cells, particularly effector T cells, need to expand rapidly when immune response is activated. Treatments that target rapidly proliferating cancer cells can therefore reduce the numbers and potency of anti-tumor effectors. Potential approaches to inhibit glycolysis, nucleotide metabolism, glutamine or arginine should thus be carefully evaluated for their impact on T cells. In contrast, therapies that limit Tregs (targeting OXPHOS and FA metabolism mostly) have a very good potential to improve outcomes of therapy.

Like immune cells, ECs also proliferate during tumor angiogenesis and rely mostly on glycolysis. An outright elimination of TECs is not warranted, as this impairs vessel function, promotes tumor hypoxia and can potentially induce metastases. On the other hand, partial inhibition of pro-angiogenic metabolic pathways such as glycolysis that reduces EC proliferation and angiogenic activity, leads to vessel normalization, increased perfusion and better response to combinatorial therapy.

Targeting CAFs with current metabolic treatment seem to have the least negative effects, as there is little evidence that CAFs would decrease effectiveness of existing metabolic drugs. Instead, these drugs tend to reduce nutrients and the overall support that CAFs provide to cancer cells. Proline synthesis seems to be a particularly promising target in CAFs, yet at present there are only limited options for pharmacological targeting of this pathway.

What is the best way forward? We see considerable potential in combinatorial metabolic therapy. Agents targeting OXPHOS and FA metabolism seem to show the most benefits in the TME and may thus be good candidates for such combinations. Furthermore, metabolic drugs often target fundamental metabolic processes that are shared across cell types. It might therefore be convenient to focus on auxiliary components of these pathways such as plasma membrane transporters that are more cell type specific, allowing differentiation between cancer cells and for example T cells. A prime example of such strategy is the glutamine uptake inhibitor V-9302, in response to which effector T cells, but not cancer cells, upregulate an alternative transporter making them insensitive. In the long run, future treatments would benefit from design of compounds that are less easily taken up by certain TME cell types such as effector T cells. Similarly, engineered CAR-T cells could in principle be designed as resistant to metabolic inhibitors.

To sum up, when carefully considered, finetuning the effects of metabolic treatments in the TME represents a promising opportunity for new metabolic anti-cancer strategies.

## Author contributions

PH prepared figures. All authors prepared and revised the manuscript and approved the final version.

## Glossary

**Table:**

2-DG	2-deoxyglucose
2HG	(R)-2-hydroxygultarate
3PO	3-(3-pyridinyl)-1-(4-pyridinyl)-2-propen-1-one
5-FU	5-fluorouracil
6-MP	6-mercaptopurine
6-TG	6-thioguanine
AAs	amino acids
ACC	acetyl-CoA carboxylase
ADI	arginine deiminase
αKG	α-ketoglutarate
ASNS	asparagine synthase
ASS	argininosuccinate synthase
BPTES	bis-2-(5-phenylacetamido-1,2,4-thiadiazol-2-yl)-ethyl sulfide
CAFs	cancer associated fibroblasts
CAR-T	Chimeric antigen T cells
CHC	a-cyano-4-hydroxycinnamate
CI	respiratory complex I
CPT1	carnitine O-palmitoyltransferase 1
DCA	dichloroacetate
DCs	dendritic cells
DHODH	dihydroorotate dehydrogenase
DON	6-diazo-5-oxo-L-norleucine
ECs	endothelial cells
FAs	fatty acid
FAO	fatty acid β-oxidation
FGF	fibroblast growth factor
FASN	fatty acid synthase
FH	fumarate hydratase
GLS	glutaminases
GLUD	glutamate dehydrogenase
GLUTs	glucose transporters
GS	glutamine synthase
HK2	hexokinase 2
IDH	isocitrate dehydrogenase
LDHA	lactate dehydrogenase A
MCT	monocarboxylate transporters
NK	natural killer cells
OXPHOS	oxidative phosphorylation
PDK	pyruvate dehydrogenase kinase
PFK-1	phosphofructokinase 1
PFKFB3	6-phosphofructo-2-kinase/fructose-2, 6-biphosphatase 3
PD-L1	programmed death ligand-1
PPAR	peroxisome proliferator–activated receptor
PRAT	5-phosphoribosyl-1-pyro-phosphatase amidotransferase
SDH	succinate dehydrogenase
SLC1A5	solute carrier family 1 member 5
SUCL	succinyl-CoA ligase
TAMs	tumor-associated macrophages
TCA	tricarboxylic acid
TECs	tumor endothelial cells
TME	tumor microenvironment
TOFA	5−tetradecyloxy−2−furoic acid
Treg	regulatory T cells
VEGF	vascular endothelial growth factor
VEFGR2	vascular endothelial growth factor receptor 2
